# The Medical, Surgical, and Hygienic Exhibition, 1901

**Published:** 1901-06-15

**Authors:** 


### THE MEDICAL, SURGICAL, AND HYGIENIC EXHIBITION, 1901.

(*Continued from page* 169.)

Last week we dealt with the drugs and surgical appliances which were displayed at this exhibition. We now proceed to the various disinfectants and sanitary appliances and to the foods, which were exhibited in great variety.

Messrs. Newton Chambers and Co., Limited, Thorncliffe, near Sheffield, had on view their powerful and effective Izal disinfectants, enormous quantities of which have been supplied for the Army in South Africa; Medical Izal, which is a strong antiseptic emulsion for purely medical purposes and recommended for obstetrical work, Izal Perles (globules containing Izal oil for internal antisepsis), and other Izal preparations and soaps. The Izal disinfectants are non-poisonous.

The Jeyes Sanitary Compounds Company, Limited, 64 Cannon Street, E.C., were prominent with Jeyes' Fluid, a coal tar disinfectant, Creolin (a refined preparation of Jeyes' Fluid for medical purposes), Creolin Dusting Powder, and "Branalcane," a preparation for local application in diphtheria and throat affections. They also were showing an excellent perfumed coal tar soap, and other toilet preparations.

Messrs. A. Boake, Roberts and Co., Limited, Stratford, E., gave an exhibit of their well-known Sulphur Dioxide ($SO_2$) for fumigation, safely stored in hermetically sealed vessels, and Lozar, a fluid preparation of phenol, which is an antiseptic, disinfectant, and deodorant. They also had a brilliant ammonia, which should find favour for toilet purposes.

Messrs. J. Defries and Sons, Limited, 146 Houndsditch, E.C., exhibited the Pasteur (Chamberland) filters, which have been largely adopted at institutions and private houses. They also showed their "Equifex" disinfectant appliances. The Berkefeld Filter Company, Limited, 121 Oxford Street, W., showed in operation their pressure filters for hospital and household use. These filters, which are rapid in action, are simple in construction and can easily be sterilised.

Messrs. James Keith and Blackman Company, Limited, 27 Farringdon Avenue, E.C., had in operation their electric fan and motor combined, for ventilating hospitals and other public buildings.

Messrs. Ewart and Son, Limited, 346 Euston Road, N.W., had an exhibit of their well-known Geysers for the production of hot water at short notice, and other fittings for bath rooms.

The sanitary appliances and fittings, designed for use in hospitals, were a prominent feature of this exhibition, and much interest was taken in the mechanism for working the water supply and waste by treadle arrangement, by which the hands are left free. Messrs. Doulton and Co., Lambeth, S.E., showed the following, fitted with this pedal action:—A bed-pan and urine bottle sink of white glazed ware, a special sink for bed-pans, and a handsome earthenware lavatory for operating rooms. They also had a vitreous enamelled operating table, so arranged as to be moved to any position. The exhibit of Messrs. George Jennings, Limited, Lambeth Palace Road, included sinks, w.c. apparatus, and other sanitary appliances; a syphonic discharge w.c. with chair seat, sitz jet, and spray; and an independent tubular bath with hot and cold shower, wave, douche, needle, and sitz sprays. Messrs. Tylor and Sons, Limited, 2 Newgate Street, E.C., had on view an operating theatre lavatory, and their "Regal" lavatory—both with pedal action, and cantilever bracket water closets, which, being fitted clear of the floor, are accesible for cleaning purposes.

The importance of milk as an article of diet was amply demonstrated at this exhibition, and examples were in evidence of the purity and high quality which has been attained. The Aylesbury Dairy Company, Limited, St. Petersburgh Place, Bayswater, W., had an extensive display of milk preparations and specimens illustrating their researches on the composition of human milk compared with the milk of the cow, goat, ass, and mare. Attention was specially drawn to their scheme for supplying hospitals, which provides for the inspection of the farm, the cow, and the water supply. The milk is analysed daily, and thus a report on any given supply can be made direct to hospital officials when required. Messrs. Welford and Sons, Limited, Elgin Avenue, Maida Vale, W., exhibited specimens of various milk preparations, particular attention being directed to their asses' milk and koumiss, which are specialities of this firm. Messrs. Welford undertake to execute orders for medical prescriptions of milk in the required proportion of fat, milk, sugar, proteids, &c. Mr. Henri Nestlé, 48 Cannon Street, E.C., had a large display of Nestlé's Food, Condensed Swiss milk, and Viking milk—a condensed milk without sugar. The products of this firm are considered of a high order, while the Nestlé's Food, which was the principal feature of the exhibit, is held in much esteem as a diet for infants.

(*To be continued.*)

